# Supplementary material for: Characterization of a New Stripe Rust Resistance Gene on Chromosome 2StS from Thinopyrum intermedium in Wheat
Source: Plants (Basel). 2025 May 20;14(10):1538. doi: 10.3390/plants14101538 (PMC12114733; doi:10.3390/plants14101538)
Supplement: Supplementary file 1 [file plants-14-01538-s001.zip › plants-3646022-supplementary.pdf]

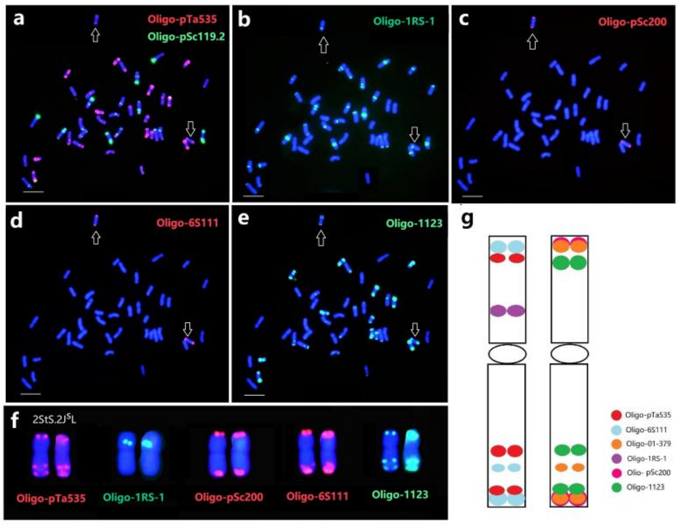

Figure S1. Sequential ND-FISH patterns of wheat-*Th. intermedium* partial 2StS.2J^{S}L (2D) disomic substitution line Th93-1-6. The probes Oligo-pSc119.2 (green) + Oligo-pTa535 (red)(a), Oligo-1RS-1 (green)(b), Oligo-pSc200 (red)(c), Oligo-6S111 (red)(d) and Oligo-1123 (green)(e) are presented, respectively. The cut and pasted chromosomes are list in (f). The diagram indicates the hybridization sites of different probes on chromosomes 2StS.2J^{S}L(g).

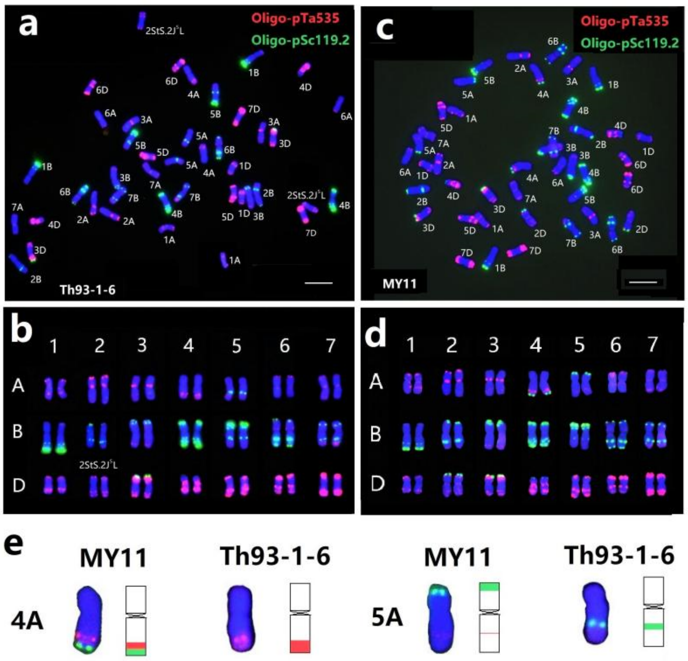

Figure S2. ND-FISH of mitotic metaphase of wheat parents Th93-3-6 and MY11 by probes Oligo-pTa535 (red) + Oligo-pSc119.2 (green). The karyotype for Th93-1-6 (a, b), MY11 (c, d) are shown.

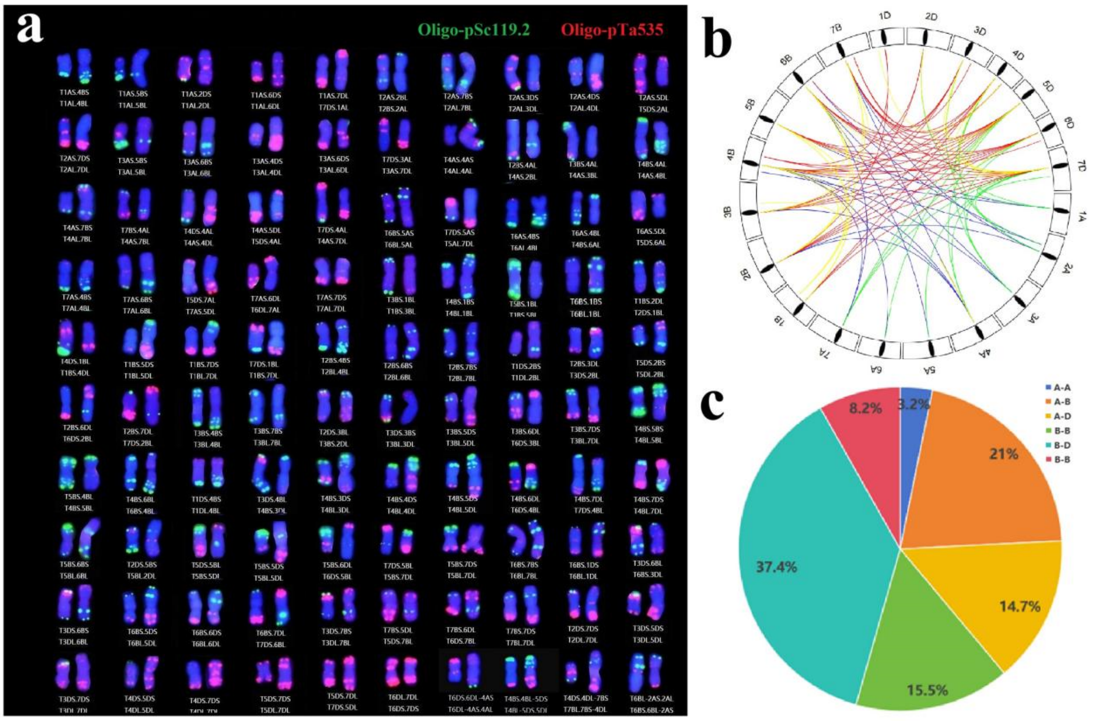

Figure S3. (a) The typical FISH karyotypes of chromosomal rearrangements in wheat. (b) The profile of 537 translocation events among A, B and D subgenome. Blue, green, red and yellow lines indicated the translocations occurred in the A-B, A-D, B-D and the same genome (A-A, B-B and D-D), respectively. (c) The frequencies of translocations between wheat sub-genomes.

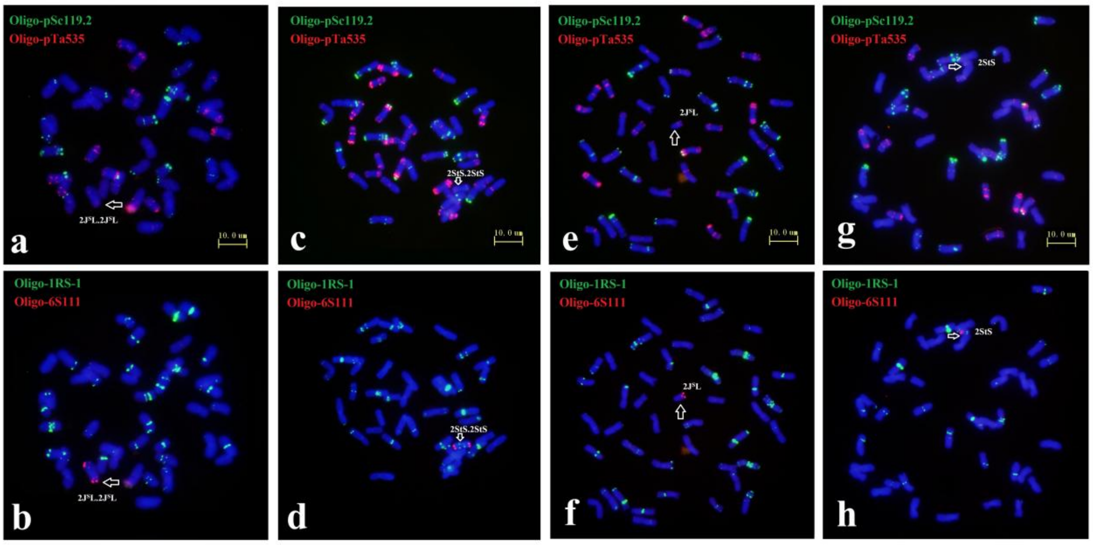

Figure S4. Sequential ND-FISH of $2J^{S}L.2J^{S}L$ (a, b), 2StS.2StS (c, d), $2J^{S}L$ telosomes (e, f) and 2StS telosomes (g, h). The probes for FISH are: Oligo-pTa535 + Oligo-pSc119.2 (a, c, e, g), Oligo-1RS-1 + Oligo-6S111 (b, d, f, h). Arrows indicated chromosomes $2StS.2J^{S}L$ aberrations.

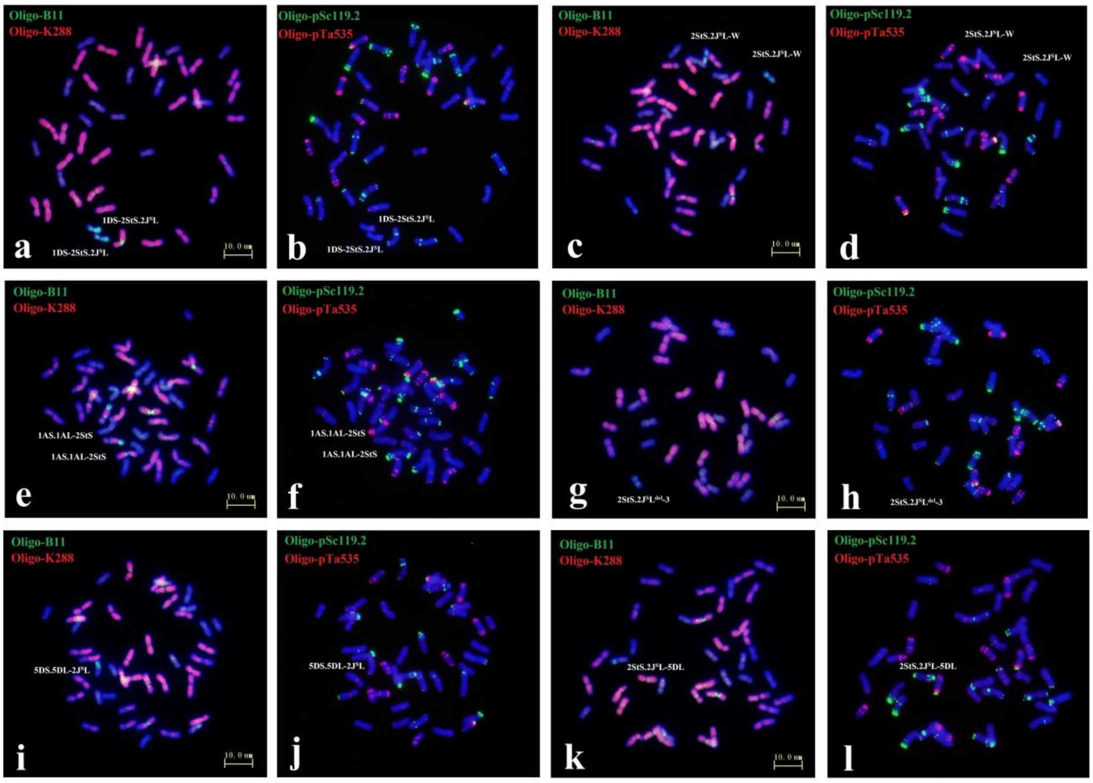

Figure S5. Sequential FISH for eight 2StS.2J^{S}L aberrations with probes Oligo-k288 + Oligo-B11 (a, c, e, g, i, k), Oligo-pTa535 + Oligo-pSc119.2 (b, d, f, h, j, l). They were line C651: 1DS-2StS.2J^{S}L (a, b), line C788: 2StS.2J^{S}L-W (c, d), line C42: 1AS.1AL-2StS(e, f), line C269: 2StS.2J^{S}L^{del}-3 (g, h), line C796: 5DS.5DL-2J^{S}L (i, j), line C797: 2StS.2J^{S}L-5DL (k, l).

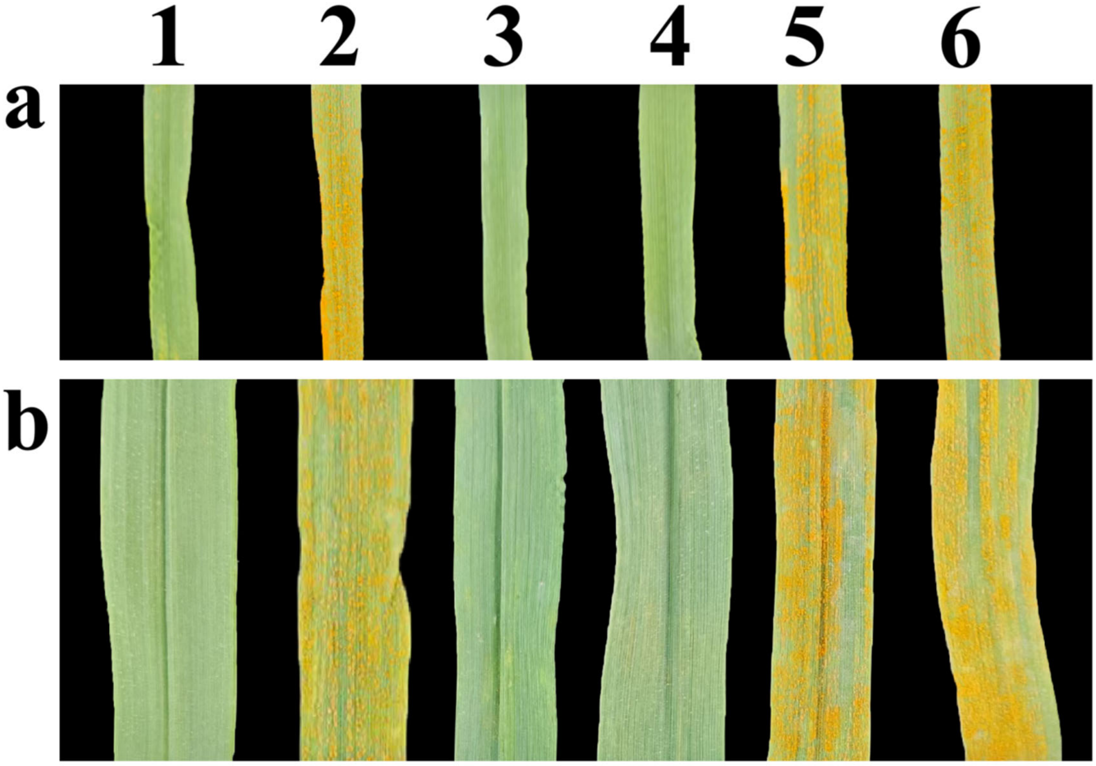

Figure S6. Stripe rust responses of Th93-1-6, wheat cultivar MY11 and their progenies at (a) seedling stages and (b) adult plant stage. 1,Th93-1-6; 2, MY11; 3, 2StS telosomes carrier; 4,

iso-telosomic 2StS.2StS carrier; 5, $2J^{S}L$ telosomes carrier; 6, iso-telosomic $2J^{S}L.2J^{S}L$ carrier.

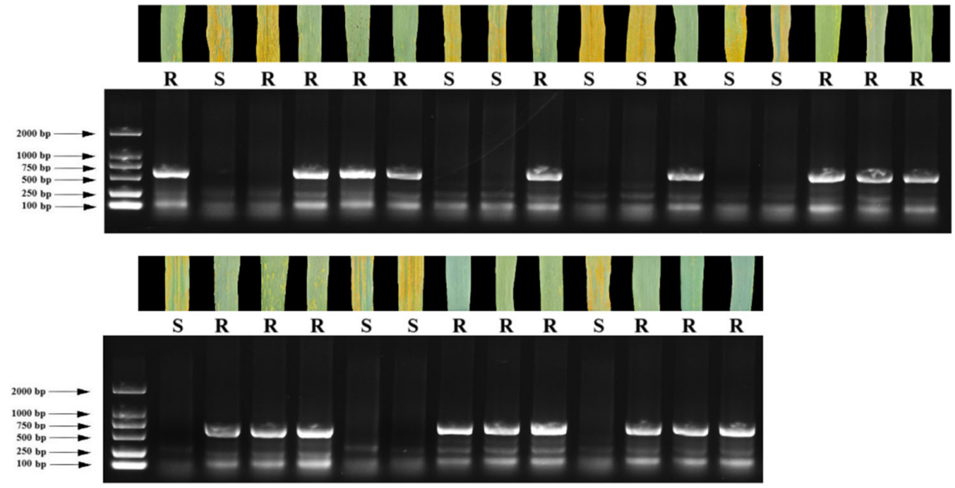

Figure S7. Stripe rust reactions and amplification results of marker 2St_103 in 30 monosomic 2StS telosomes addition progenies.

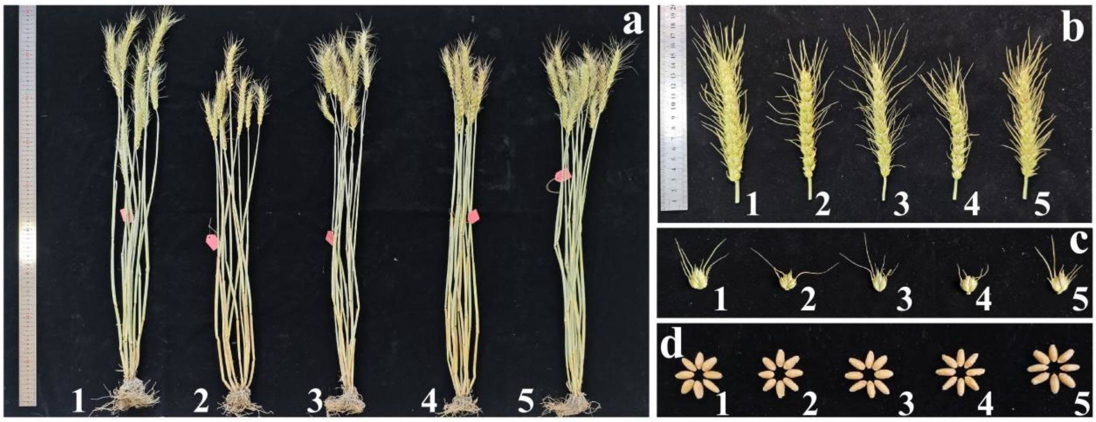

Figure S8. Plant morphology of 1, Th93-1-6; 2, MY11; 3, 2StS carrier; 4, $2J^{S}L$ carrier; 5, C152.

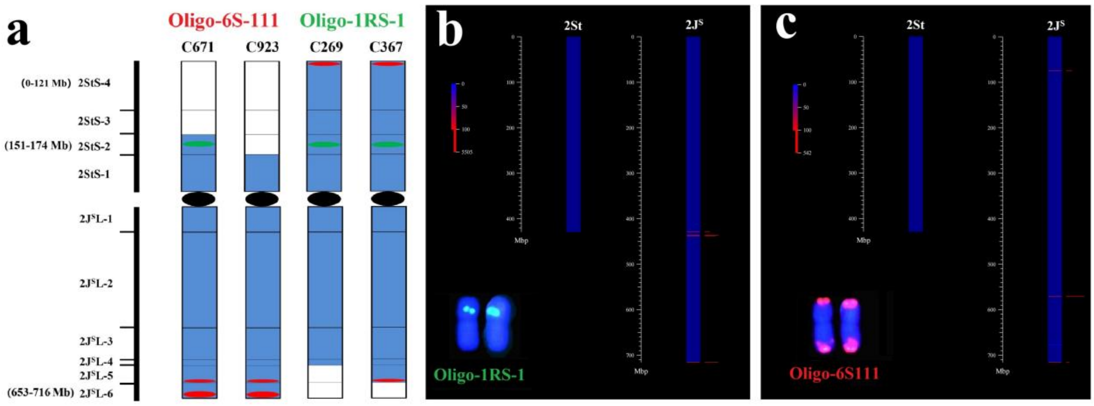

Figure S9. The ND-FISH results and physical distribution of Oligo-1RS-1 and Oligo-6S111 in chromosomes of $2StS.2J^{S}L$. (a) The hybridization sites of probes Oligo-1RS-1 and Oligo-6S111 in four $2StS.2J^{S}L$ aberration lines. (b-c) The prediction of Oligo-1RS-1 and Oligo-6S111 in *Thinopyrum intermedium* genome V.2.1 used default parameters for the blast and filter steps according to the website B2DSC.

Table S1 The sequence of Oligo probes used in our study

Oligo probes	Sequences	Reference
Oligo-pTa535	AAAAACTTGACGCACGTCACGTACAAATTGGACAAACTCTTTCGGAGTAT CAGGGTTTC	[54]
Oligo-pSc119.2	CCGTTTTGTGGACTATTACTCACCGCTTTGGGGTCCCATAGCTAT	[54]
Oligo-B11	TCCGCTCACCTTGATGACAACATCAGGTGGAATTCCGTTCGAGGG	[55]
Oligo-k288	CTTCATAGTCCGGGAGTCCGGCCAAAGGTCATAGTCCG GCCATCC	[44]
Oligo-1RS-1	ACAGGGTGGCATGGTGTCACGTGCTCGTCTCGGCGTCCGTCGTCGC	[56]
Oligo-pSc200	CTCACTTGCTTTGAGAGTCTCGATCAATTCGGACTCTAGGTTGATTTTTGTA TTTTCT	[57]
Oligo-6S111	AGGACTTCTAATTTTTATGATCTGGATGATTTTACATACGCGCACACACCCA AGCAC	[58]
Oligo-pDb12H	TCAGAATTTTTAGGATAGCAGAAGTATTCGAAATACCCAGATTGCTACAG	[59]

Table S2 The developed marker sequences for 2StS chromosomes

Makers	Forward	Reverse
2St_99	CTCGGACTCGGATTATAGCT	CCATAGAGCGTATCACCAGA
2St_102	AGCTGGTACTAATCGGAGTC	CTGATCGGTATGTAGTCCCT
2St_103	CTGGCTGTCTCAAGATTGTG	AGCAATGAGGTAACGGTCTG
